# Classification and Prediction on Hypertension with Blood Pressure Determinants in a Deep Learning Algorithm

**DOI:** 10.3390/ijerph192215301

**Published:** 2022-11-19

**Authors:** Hyerim Kim, Seunghyeon Hwang, Suwon Lee, Yoona Kim

**Affiliations:** 1 Department of Food and Nutrition, Gyeongsang National University, Jinju 52828, Republic of Korea; 2 Department of Computer Science, Gyeongsang National University, Jinju 52828, Republic of Korea; 3 Department of Computer Science, The Research Institute of Natural Science, Gyeongsang National University, Jinju 52828, Republic of Korea; 4 Department of Food and Nutrition, Institute of Agriculture and Life Science, Gyeongsang National University, Jinju 52828, Republic of Korea

**Keywords:** deep learning, deep neural network, hypertension, decision tree, nutrient and dietary pattern, energy intake adjustment

## Abstract

Few studies classified and predicted hypertension using blood pressure (BP)-related determinants in a deep learning algorithm. The objective of this study is to develop a deep learning algorithm for the classification and prediction of hypertension with BP-related factors based on the Korean Genome and Epidemiology Study-Ansan and Ansung baseline survey. We also investigated whether energy intake adjustment is adequate for deep learning algorithms. We constructed a deep neural network (DNN) in which the number of hidden layers and the number of nodes in each hidden layer are experimentally selected, and we trained the DNN to diagnose hypertension using the dataset while varying the energy intake adjustment method in four ways. For comparison, we trained a decision tree in the same way. Experimental results showed that the DNN performs better than the decision tree in all aspects, such as having higher sensitivity, specificity, F1-score, and accuracy. In addition, we found that unlike general machine learning algorithms, including the decision tree, the DNNs perform best when energy intake is not adjusted. The result indicates that energy intake adjustment is not required when using a deep learning algorithm to classify and predict hypertension with BP-related factors.

## 1. Introduction

The prevalence of hypertension is estimated in 30–50% of individuals aged 40–79 years in global populations of the following 12 high-income countries: Australia, Canada, Finland, Germany, Ireland, Italy, Japan, New Zealand, South Korea, Spain, the UK, and the USA [1]. In South Korea, hypertension was prevalent in 29% of individuals aged over 30 years [2]. Approximately 1 billion people globally have hypertension, influencing 10.4 million mortalities in 2017 as a leading cause of death [3]. One-third of adults globally were reported to have hypertension [4]. Hypertension promotes the risk of cardiovascular disease (CVD), heart disease, type 2 diabetes mellitus (T2DM), brain disease, and kidney disease [5,6]. Hypertension contributed to approximately 22.3% of CVD morbidity and mortality [7].

The elevated prevalence of hypertension is considered a global public health issue by the World Health Organization (WHO) [8,9,10]. The WHO recommends lifestyle modifications and antihypertensive medications for the prevention and treatment of hypertension [8,9,10]. Family history, age, co-existing diseases, and so on are non-modifiable factors. Diet, physical activity, smoking, alcohol consumption, and so on are modifiable factors. Lifestyle modifications independent of antihypertensive medication is encouraged for hypertension management [11,12,13]. Modifiable factors, especially a diet, may lower risk of hypertension. The Dietary Approaches to Stop Hypertension (DASH) diet is recommended for blood pressure (BP) reduction. The DASH diet is rich in fruits, vegetables, low-fat dairy, and whole grains, and low in saturated fat, red and processed meats, and sugar [14,15,16]. Dietary patterns high or/and low in certain nutrients were associated with a reduction in BP [17,18,19]. 

Recently, as a new strategy for blood pressure management, the importance of predicting blood pressure by using machine learning (ML) of big data-based artificial intelligence (AI) methods has been highlighted [20].

Deep learning, a part of machine learning algorithms, progressively performs new higher-level tasks from a test dataset through artificial neural network (ANN) with multiple layers after learning by itself using a training dataset [21,22]. Deep learning architectures include a deep neural network (DNN), a deep belief network, a stacked autoencoder, a convolutional neural network, and a recurrent neural network [21,22]. A DNN is an extended ANN comprising multiple layers of linear and non-linear operations between the input and output layers. A DNN enables the algorithm to learn complicated representations of the input data [21,22,23].

Few studies developing prediction algorithm of cardiometabolic disease with dietary factors [24,25,26,27,28] or without dietary factors [29,30,31] exist in the machine learning algorithm. So far, a study on the cardiometabolic disease risk prediction algorithm, including nutritional intakes using DNN, exists [32].

Therefore, the aims of this study were as follows: (a) to develop and evaluate a deep learning algorithm for hypertension classification and prediction with BP determinants in the general Korean adult population using the Korean Genome and Epidemiology Study (KoGES) baseline survey; (b) to investigate to what extent energy intake adjustment affects hypertension classification and prediction in a developed deep learning algorithm.

The rest of this paper is organized as follows: Section 2 describes materials and methods related to this study; Section 3 analyses the independent variables and the decision tree for variable importance; and Section 4 presents the experimental results for the proposed method for building DNN, and the decision tree was compared for four datasets. Finally, the paper concludes in Section 5 and Section 6, and seeks future research directions.

## 2. Materials and Methods

**Figure 1:**
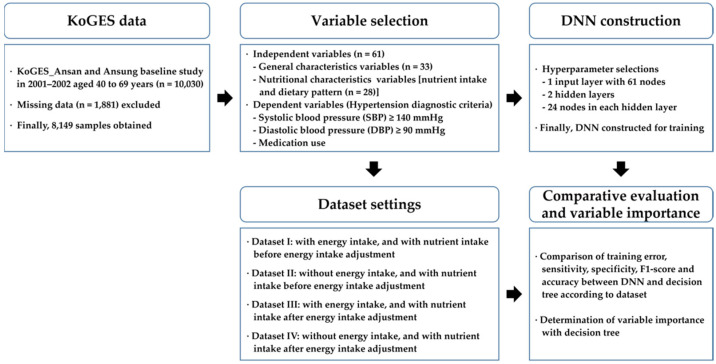
A flowchart for research process.

A flowchart for the research process is presented in Figure 1. From the KoGES baseline survey, a dataset consisting of 8149 samples with 61 independent variables and a class label, is obtained through variable selection. From the dataset, a DNN with experimentally selected hyperparameters are prepared. Then, four datasets are set according to the energy intake adjustment method, and a DNN is trained and evaluated for each dataset. A decision tree is used for comparative evaluation, and variable importance is analyzed using the decision tree.

### 2.1. Data and Subjects

This study was performed using epidemiological data obtained from the population-based cohorts in the KoGES-Ansan and Ansung baseline study in 2001–2002. The KoGES has been conducting health and lifestyle-related surveys and examinations since 2001, targeting the general population aged 40 to 69 years by the National Institutes of Health at the Korea Centers for Disease Control and Prevention. The KoGES is a large-scale cohort data that collects human materials such as blood and urine DNA and conducts follow-up studies [33,34,35]. The general population-based cohorts include community-based cohorts (Ansan and Ansung), urban-based cohorts and rural-based cohorts. The gene environment model cohorts include twin and familial cohorts, the immigrant cohorts, and emigrant cohorts (Japan and China) [33,34,35]. The semi-quantitative food frequency questionnaire (FFQ) of KoGES was developed by extracting high-frequency foods from Korean men and women aged 40–69 years based on the Korea Health and Nutrition Examination Survey (KHANES) [36]. Foods were selected up to a level where the cumulative proportion of contributing foods was 90% [37,38]. Two FFQs (twice at a 1-year interval) and 12-day diet records (DRs) with 3 days during each of the 4 seasons for 124 people were collected for validation and reproducibility. A comparison of nutrients intake from DRs with two FFQs was performed [39].

The KoGES-Ansan and Ansung study consisted of baseline study recruited in 2001–2002 and an 8th follow-up study. This study used 10,030 samples from community baseline cohorts (Ansan and Ansung study) among the general population-based cohorts recruited in 2001–2002 [33,34]. The variables of height and weight were replaced with the group mean. The variable of alcohol intake was replaced with mode. Other missing data were excluded. Finally, 8149 samples were included as a dataset.

The study was conducted according to the guidelines of the Declaration of Helsinki. Written informed consent was obtained from all subjects participated in this study. The KoGES-Ansan and Ansung study was approved by the Ethics Review Committee of the Korean Health and Genomic study at the Korea National Institute of Health (2018-03-05-5C-A). The present study protocol was approved by the Ethics Review Committee of Gyeongsang National University (KHGIRB-19-398).

### 2.2. Variable Selection 

We attempted to include as many variables as possible related to hypertension risk factors [40,41,42,43]. Finally, 61 independent variables were included, such as: general characteristics [n = 33; e.g., sex, age, body mass index (BMI), waist circumference, education, income, smoking and drinking], nutrient intakes (n = 23), and dietary patterns (n = 5).

Five dietary patterns were classified by a factor analysis as suggested elsewhere [44]. Briefly, a factor analysis was conducted as follows: 103 food items were categorized into 17 food groups (Table 1). The daily intake amount of each food group was applied to the principal component method. As shown in Table 2, a factor analysis was used for the extraction of the food group. Verimex with Kaiser–Meyer–Olkin (KMO) normalization was performed for factor rotation. The factor rotation converges at the 7th time in performing the iterative calculations. The KMO measure of sampling adequacy was 0.77, which was more than 0.7. Bartlett’s test of sphericity was significant with *p* < 0.005. The factor analysis was considered to be appropriate with these indicators of Bartlett’s sphericity test and the KMO index. The types of food groups were used with absolute values of factor loadings of ≥0.3 to identify factor characteristics.

The diagnostic criteria for hypertension were treated as dependent variables. Hypertension was defined as either increased BP [defined as systolic blood pressure (SBP) ≥ 140 mmHg or diastolic blood pressure (DBP) ≥ 90 mmHg] or antihypertensive medication use. Systolic and diastolic BP was measured twice in a supine position based on a standardized protocol. The hypertension diagnosis was treated as dependent variables. Hypertension was set as ‘1’, and normal cases were set as ‘0’ (Table 3).

### 2.3. Statistical Analysis 

Categorical variables were cross-analyzed to understand the distributional charac-teristics of general characteristic variables and nutritional characteristics variables ac-cording to hypertension and non-hypertension. A chi-squared analysis was performed for categorical variables. Continuous independent variables were tested for normality. The Kolomogorov–Smirnov test, Q-Q plots, and histograms were used to test for the normality of distribution. Mann–Whitney U analysis was performed as they were not normally distributed after log transformation. Continuous independent variables analyzed by Mann–Whitney U analysis are presented as medians and interquartile ranges. Statistical analysis was performed with SPSS 27.0 (IBM, Chicago, IL, USA).

### 2.4. Development Environment

Keras (version 2.8.0) of TensorFlow (version 2.8.2) was used to build a DNN in Python (version 3.7.14). The deep learning algorithm was implemented in the Colaboratory (version 18.04.5) environment provided by Google. We also used SimpleImputer in the scilkit-learn module (version 1.0.2) for data pre-processing.

### 2.5. Data Pre-Processing

As the first pre-processing, we handled the missing values. Samples with missing values in categorical variables were excluded from the data. Missing values of continuous variables were filled in with the mean value using SimpleImputer.

As the second pre-processing, we performed data encoding and data scaling. For data encoding, one-hot-encoding was applied to categorical variables. The values of continuous variables were scaled by data normalization, data standardization, and data quantile transformer (quartile data) methods. Data scaling is a process of data pre-processing to prevent the case where the deep learning algorithm converges to 0 or diverges to infinity during the learning process because the learning does not proceed smoothly when the data value is too large or too small.

To solve the unbalanced data problem resulting from the difference in the amount of positive and negative samples in the training stage, we oversampled the data using the synthetic minority oversampling technique (SMOTE), a heuristic method for oversampling. SMOTE oversamples the data by the following procedure. First, a sample belonging to the minority class is selected. Then, the k samples closest to the selected sample in feature space are found using the k-nearest neighbors (k-NN) algorithm, a non-parametric supervised learning method used for classification or regression. A random point on the vector between one of those k samples and the first selected sample is created as a synthetic sample. By oversampling, the positive samples that were lacking in the training stage were sufficiently retained.

### 2.6. Dataset Setting

To find out how energy intake adjustment affects the results, four datasets were defined by different energy intake adjustment methods as shown in Table 4. Energy intake adjustment was performed by calculating nutrient intake per 1000 Kcal as the most nutrient intake was considered to be proportional to the total calorie intake per day. 

### 2.7. Algorithm Construction

**Figure 2:**
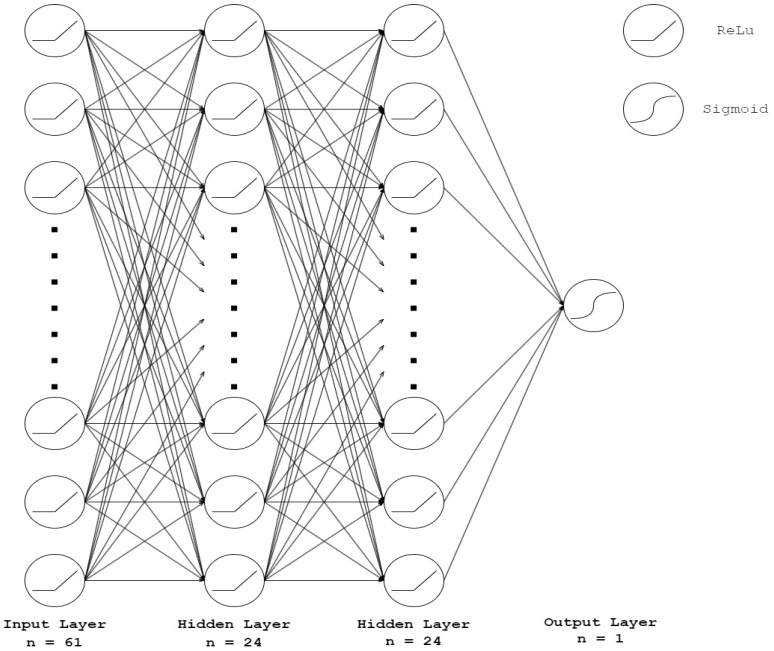
An example of the structure of the DNN we constructed.

We selected a DNN as a deep learning architecture and constructed the DNN by varying the number of hidden layers from 2 to 5 and the number of nodes belonging to each hidden layer from 8 to 64. The input layer consisted of 61 nodes as the number of variables. The output layer had one node for 2-class classification and applied sigmoid as an activation function. The rectified linear unit was applied as activation function to each hidden layer to prevent vanishing gradient. The connection of each layer was dropped out with a probability of 0 to 0.5 to prevent overfitting. The DNN is trained using the Adam optimizer [45], which combined the pros of Adagrad [46] and RMSprop [47]. Figure 2 shows one of the best performing DNN we constructed.

### 2.8. Decision Tree Processing for Variable Importance

A decision tree is a method of inductive reasoning by expressing decision rules (logical sum, logical product) in a tree structure, and by analyzing the entire data into several small groups [48]. We used the decision tree not only for comparison with the results of the deep learning algorithm but also to figure out the variable importance that is difficult to figure out with the deep learning algorithm. Variable importance was identified by figuring out variables with low Gini impurity with the CART algorithm using 61 variables by expressing decision rules (logical sum, logical product) in a tree structure. A decision tree was created by figuring out the variables that minimizes the impurity among 61 variables. Variable importance was determined without setting the maximum depth of the tree. In the decision tree, a variable was not used when the variable importance was 0. A variable was used when the variable importance was 1, indicating that a decision tree precisely predicted variable importance. The variable was regarded invalid when the variable importance was lower than that of child nodes. The variable was considered valid when the variable importance was larger than that of child nodes. Finally, the decision tree was converged to one result.

## 3. Analysis

### 3.1. Baseline Characteristics of Independent Variables

The baseline characteristics of independent variables are summarised in Table 5. The data characteristics were analyzed with hypertension, non-hypertension training dataset, and testing dataset. The characteristics of the independent variables of the subjects were analyzed according to hypertension (n = 1883) and non-hypertension (n = 6266). A total of 8149 data (3850 males and 4299 females) of the KoGES-Ansan and Ansung baseline study in 2001–2002 were randomly modelled in an 8:2 ratio for training dataset and testing dataset as shown in Table 5. 

The number of men with hypertension was 858 (45.6%), while women with hypertension was 1025 (54.4%) (*p* = 0.096). The prevalence of hypertension showed a tendency to increase with age (*p* < 0.001). BMI and waist circumference were higher in subjects with hypertension compared with those without hypertension (*p* < 0.001). 

The prevalence of hypertension was higher in subjects who graduated from elementary school than those who graduated from middle school, high school, and university (n = 875; 46.5%). More than half of the subjects answered that they had never smoked, regardless of whether or not they had hypertension (*p* < 0.001). Among subjects with hypertension, 922 subjects (49%) answered that they currently drink (*p* = 0.037) (See Table S1 for the detailed data). The mean energy intake of hypertensive subjects was 1771 Kcal (median = 1464), and that of non-hypertensive subjects were 1849 Kcal (median = 1545).

In the training dataset and testing dataset, the mean body weight was 63.2 kg, and the mean waist circumference was 82.4 cm. The average energy intake of each man and woman was 1937 Kcal in this study. The estimated energy need (EER) for Koreans in 2020 were 2233 Kcal and 1733 Kcal for men and women, respectively, aged between 40 and 69 years old. The men consumed a lower intake of about 300 Kcal than the EER. The women consumed about 200 Kcal higher than the EER. The recommended protein intakes for Koreans in 2020 were 61.6 g and 50 g for men and women, respectively. The recommended daily protein intake of subjects was on average 64.12 g, which was higher than the recommended amount. According to the 2020 energy intake standards for Koreans, fat was an appropriate ratio of energy intake and a sufficient intake of 15–30% was set. According to the standard, intake of 32.6 g to 65.8 g was sufficient. Subjects consumed 32.31 g of fat, which was about 15% of the total energy intake. The mean daily recommended intake of carbohydrates for Koreans was 130 g, and subjects consumed 340.56 g of carbohydrate which was 2.6-times higher. The subjects of this study consumed most of their energy with carbohydrates. Calcium was ingested at 476.54 mg per day. The recommended intake of calcium was 750 mg for men and 766 mg for women. In this study, calcium intake was at a level of 63% of the recommended intake. The mean daily recommended intake for phosphorus was 700 mg, and subjects consumed 1021.27 mg or more. The subjects consumed the recommended intake level of 10.86 mg of iron. The mean daily recommended intake of iron was 9.6 mg for men and 10 mg for women. 

The mean daily intake of potassium was 2521.69 mg. potassium intake was less than the sufficient intake of 3500 mg. The mean daily intake of retinoid was 533.27 R.E. The mean daily intake for men was 750 R.E for men and for women, 626.6 R.E., which was 77% of the recommended intake. Sodium was ingested 3169.46 mg. Both men and women had an adequate intake (AI) of 1433 mg, which was more than 2.2 times higher, and sodium intake for chronic disease risk reduction (CDRR) was 2233 mg, which was 1.4 times more. The recommended intake of thiamine was 1.16 mg for men and 1.1 mg for women. The subjects consumed on average 1.25 mg of thiamine. The recommended intake for riboflavin was 1.03 mg, and niacin (nicotinic acid) was 1.46 mg for men and 1.16 mg for women. The subjects took on average 15.56 mg of niacin (nicotinic acid). Subjects consumed 126.02 mg of ascorbic acid. The recommended intake was 100 mg of ascorbic acid. 

Zinc was taken on average 8.75 mg per day. The average daily recommended zinc intake for Koreans was 9.6 mg and 7.6 mg for men and women, which was the recommended intake for the study subjects. Pyridoaxamine was consumed at 1.78 µg. The recommended intake of pyridoaxamine was 2.4 µg, which was lower than the recommended intake for Korean nutrition in 2020. The average intake of folate was 245 µg for all data. The recommended intake of folate was 400 µg for both men and women accounting for 61% of the total intake. In this study, 68.37 µg of retinol, 2729.30 µg of carotene, 21.43 mg of ash content, 6.98 µg of fiber, and 175.39 mg of cholesterol were ingested. The detailed baseline characteristics of independent variables included in this study are described in Table S1. 

### 3.2. Factor Analysis for Food Group Determination

The dietary patterns based on factor loading values were presented in Table 2. Dietary patterns were divided into five main categories. The first group (dietary pattern 1) includes subjects who consume cereal-oriental, cereal-western, potatoes, seeds, and nuts. The first group had the highest loading values in the order of cereal-oriental, cereal-western, potatoes, seeds, and nuts. Processed grains, potatoes and nuts were involved in this group. The second group (dietary pattern 2) consisted of subjects who consume fruits, meats, fishes and seafoods. This group primarily consume high-quality protein and fruits. Meat, fishes, and seafoods had higher loading values than fruits. The third group (dietary pattern 3) was comprised of subjects who consume snacks, eggs, seaweed, milk and dairy products and beverages. The fourth (dietary pattern 4) was the group that consumes vegetables and mushrooms. Vegetarians were involved in this group. The last group (dietary pattern 5) was comprised of subjects who consume cereal-rice, legumes, and kimchi. Subjects in this group adhered to traditional Korean meal including rice, kimchi, and legumes.

The average intake by food group or dietary pattern per day was examined in order to investigate the characteristics of each group. The highest average daily amount of each food group was cereal-rice (659,88 g), followed by kimchi (171.65 g), fruits (122.30 g), milk and dairy products (87.03 g), beverage (62.18 g), vegetable (44.49g), cereal-oriental (31.52 g), legume (19.97 g), fishes and sea foods (15.48 g), meats (10.73 g), potato (8.86 g), cereal-western (6.55 g), eggs (6.40 g), snacks (4.71 g), mushroom (2.72 g), seeds and nuts (1.88 g), and seaweed (0.77 g).

This study data showed that the intake weight of cereal-rice was at least 3.8 times and as high as 856 times compared to other food groups. The intake amount of cereal-rice was 3.8 times higher than that of kimchi of the second intake amount (171.65 g), and 856 times higher than that of seaweed of the lowest intake amount (0.77 g). Dietary pattern 5 had the highest daily intake amount (829.92 g) followed by dietary pattern 3 (161.33 g), dietary pattern 2 (148.51 g), dietary pattern 1 (48.82 g), and dietary pattern 4 (47.21 g).

### 3.3. Decision Tree for Variable Importance

**Figure 3:**
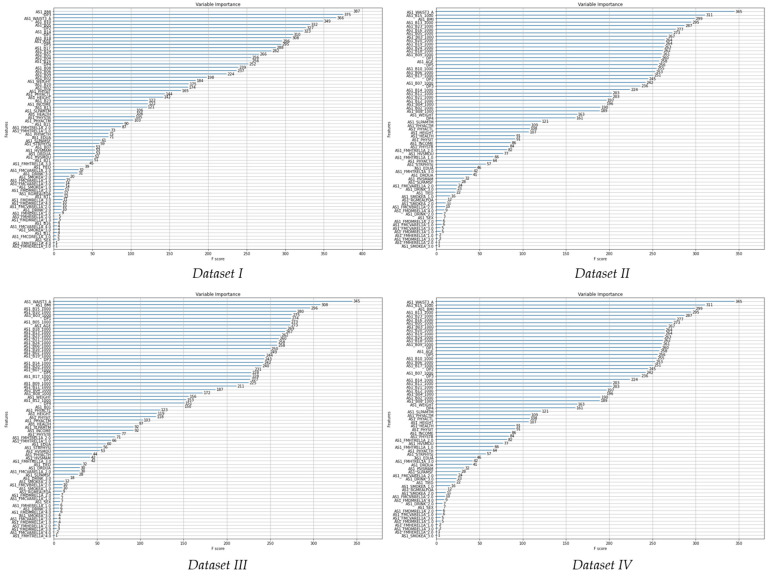
Decision tree for variable importance. AS1_age, age; AS1_BMI, BMI; AS1_B01, energy; AS1_B02, protein; AS1_B03, fat; AS1_B04, sugar (carbohydrate); AS1_B05, Ca (calcium); AS1_B06, P (phosphorus); AS1_B07, Fe (iron); AS1_B08, K (potassium); AS1_B09, vitamin A (vitamin A); AS1_B10, Na (sodium); AS1_B11, vitamin B1 (thiamine); AS1_B12, vitamin B2 (riboflavin); AS1_B13, vitamin B3 (niacin, nicotinic acid); AS1_B14, vitamin C (ascorbic acid); AS1_B15, zinc; AS1_B16, vitamin B16 (pyridoaxamine); AS1_B17, folate; AS1_B18, retinol; AS1_B19, carotene; AS1_B20, ash content; AS1_B21, fiber; AS1_B23, vitamin E (tocotrienol); AS1_B24, cholesterol; AS1_DrDuA, drinking period; AS1_Drink, can’t drink or don’t drink from the beginning (for religious reasons); AS1_EduA, education; AS1_FmCdRel1A, relationship between family members and subjects diagnosed with coronary artery disease (angina pectoris, arteriosclerosis) in the past; AS1_FmChRel1A, relationship between family members and subjects diagnosed with congestive heart failure in the past; AS1_FmCvaRel1A, relationship between family members who have been diagnosed with a stroke (paralysis) in the past and the subject; AS1_FmCvbRel1A, relationship between family members and subjects diagnosed with cerebrovascular (excluding stroke) disease in the past; AS1_FmDmRel1A, relationship between family members who have been diagnosed with diabetes in the past and the subject; AS1_FmHeRel1A, relationship between family members who have been diagnosed with heart disease in the past; AS1_FmHtRel1A, relationship between family members and subjects diagnosed with hypertension in the past; AS1_FmLpRel1A, relationship between family members and subjects diagnosed with hyperlipidemia in the past; AS1_FmPvRel1A, relationship between family members and subjects diagnosed with peripheral vascular disease in the past; AS1_Health, health status; AS1_HvSmAm, the amount of smoking per day; AS1_HvSmDu, smoking period; AS1_Income, monthly income; AS1_PhyActH, physical activity time/day (high activity); AS1_PhyActL, physical activity time/day (light activity); AS1_PhyActM, physical activity time/day (middle activity); AS1_PhySit, physical activity time/day (sedentary lifestyle); AS1_PhyStb, physical activity time/day (stable state); AS1_RgMealFqA, the number of meals a day; AS1_Sex, sex; AS1_SlpAmSf, enough time to sleep; AS1_SlpAmTm, sleeping time; AS1_SmokeA, smoking status; AS1_StrPhysJ, physical sign: the body feels drowsy and tires easily; AS1_Tied, feel tired these days; AS1_TotAlc, the total amount of alcohol consumption; AS1_WAIST3A, waist circumference average value of 3 measurement; AS1_Weight, body weight; DP1, dietary pattern 1 (cereal-oriental, cereal-western, potatoes, seeds and nuts); DP2, dietary pattern 2 (fruits, meats, fishes and seafoods); DP3, dietary pattern 3 (snacks, eggs, seaweeds, milk and dairy products, beverages); DP4, dietary pattern 4 (vegetables, mushrooms); DP5, dietary pattern 5 (cereal-rice, legumes, kimchi); Dataset I, dataset with energy intake, and nutrient intake before energy intake adjustment; Dataset II, dataset without energy intake, and with nutrient intake before energy intake adjustment; Dataset III, dataset with energy intake, and with nutrient intake after energy intake adjustment; Dataset IV, dataset without energy intake, and with nutrient intake after energy intake adjustment.

The importance between the independent variable and the dependent variable was identified through the decision tree information gain processing process (Figure 3, Table 6).

In dataset I, waist circumference (variable importance = 371) showed the highest importance followed by BMI (variable importance = 348), age (variable importance = 305), dietary pattern 3 (variable importance = 298), retinol (variable importance = 275), dietary pattern 5 (variable importance = 270), dietary pattern 1 (variable importance = 269), sodium (variable importance = 267), ascorbic acid (variable importance = 239), dietary pattern 2 (variable importance = 235).

In dataset II, waist circumference (variable importance = 358) showed the highest importance followed by dietary pattern 3 (variable importance = 322), BMI (variable importance = 321), age (variable importance = 297), sodium (variable importance = 293), carbohydrate (variable importance = 287), retinol (variable importance = 272), dietary pattern 2 (variable importance = 270), dietary pattern 1 (variable importance = 260), calcium (variable importance = 247).

In dataset III, waist circumference (variable importance = 325) showed the highest importance followed by niacin, nicotinic acid (variable importance = 285), age (variable importance = 283), dietary pattern 1 (variable importance = 266), BMI (variable importance = 265), zinc (variable importance = 253), retinol (variable importance = 242), pyridoaxamine (variable importance = 240), dietary pattern 3 (variable importance = 236), dietary pattern 5 (variable importance = 231).

In dataset IV, waist circumference (variable importance = 345) showed the highest importance followed by zinc (variable importance = 311), BMI (variable importance = 299), niacin, nicotinic acid (variable importance = 295), tocotrienol (variable importance = 287), pyridoaxamine (variable importance = 277), calcium (variable importance = 273), fat (variable importance = 267), ash content (variable importance = 264), and carotene (variable importance = 264).

In the four datasets, the variables commonly included in the top ten variables are waist circumference and BMI. In the dataset without nutrient correction, dietary pattern 1, diet pattern 2, and diet pattern 3, retinol, sodium, and age were analyzed with high variable importance. The dataset in which the nutrients were corrected to 1000 Kcal was analyzed as a variable importance with niacin, nicotinic acid, zinc and pyridoaxamine.

## 4. Experimental Results

Prior to the experiment, to select the number of hidden layers and the number of nodes belonging to each hidden layer, the number of hidden layers was varied from two to five, and the number of nodes belonging to each hidden layer was varied from 8 to 64. The results are summarized in Table 7. As shown in the Table 7, a DNN composed of two hidden layers with 24 nodes in each hidden layer that showed the best efficiency in terms of accuracy versus computational complexity was selected.

Before training and evaluating DNN, machine learning algorithms such as support vector machine, k-NN, and DT were first tested, and as a result, the three algorithms showed almost similar performance. Therefore, we report the comparative evaluation with the DT already used when analyzing variable importance among them.

The DNN and decision tree were trained with the same training dataset and tested with the same test dataset. In the process, we measured training error, sensitivity, specificity, F1-score, and accuracy for four cases of energy intake adjustment method, namely, the four datasets we defined. Table 8 presents the results of the DNN and decision tree according to the dataset. DNN derives slightly different results depending on the number of hidden layers and the number of nodes belonging to each hidden layer.

In the four datasets, the DNN showed higher sensitivity, specificity, F1-score, and accuracy compared to the decision tree. The decision tree showed the best performance using dataset III (sensitivity = 0.452, specificity = 0.761, F1-score = 0.400, accuracy = 0.690). Dataset I (sensitivity = 0.508, specificity = 0.822, F1-score = 0.482, accuracy = 0.750) showed the best performance in the DNN. In the deep learning algorithms, it seemed that the correlation of each variable was reflected in parameter learning. Dataset I, in which all variables were input without any adjustments, showed the best performance. This indicated that it is most suitable for deep learning algorithms not to adjust energy intake.

In both DNN and decision tree, the specificity was analyzed much higher than the sensitivity. In the case of sensitivity, it exceeded 0.5 only in dataset I of the DNN. This appear to be caused by the number of training data, especially the small number of positive samples for hypertension. Although oversampling was performed in the training process, the sensitivity was not improved.

## 5. Discussion

This study aimed to develop a deep learning algorithm in classification and prediction of hypertension with BP determinants based on KoGES data. The prediction accuracies of the four datasets were compared according to whether energy was included or whether nutritional intake was adjusted. In this study, dataset I (with energy intake and nutrient intake before energy intake adjustment) showed the best performance in the DNN, which means that it is most suitable for deep learning algorithms not to adjust energy intake.

Among 61 variables, waist circumference showed the highest importance. Standard body weight and abdominal circumference have an important effect on hypertension. [49]. A longitudinal study of the 1993–2015 China Health and Nutrition Survey in 11,714 individuals aged 18–66 years showed the positive association between BP and waist circumference, which was independent of BMI change [50]. 

Similar to our study, Zhao et al. 2021 [51] compared the performance of four hypertension prediction machine learning algorithms of random forest (RF), CatBoost, MLP neural network, and logistic regression (LR) with hypertension risk determinants selected by a univariate logistic regression analysis. A 10-fold cross-validation was used for model optimization. The RF performance was superior to other models. The area under the receiver operating characteristic curve (AUC), accuracy, sensitivity, and specificity on the test set was examined for the performance of four datasets. This study found that BMI, age, family history and waist circumference (WC) were key determinants for hypertension risk [51].

In this study, dietary pattern 3 consisted of milk and dairy products, snacks, beverages, eggs, and seaweed. This dietary pattern can be characterized for subjects who consumed a variety of foods. Milk and dairy products had the highest loading values (0.612), while seaweed had the lowest loading values (0.377) compared to other foods contained in this group.

We also found dietary pattern 5, which consisted of cereal-rice, kimchi, and legumes, was characterized with high in calorie intake due to a high consumption of rice and sodium attributable from kimchi consumption. A meta-analysis of randomized controlled trials (RCTs) showed that a dietary pattern rich in carbohydrate was associated with increased BP compared with a dietary pattern rich in monounsaturated fatty acids (MUFA) [52]. Kim et al. 2012 [53] also showed the positive association between the white rice and kimchi pattern and risk of obesity in Korean adults after adjustment for age, sex, energy intake, alcohol intake, smoking status, physical activity, and chronic diseases [53].

AlKaabi et al. 2020 [40] developed machine learning models for hypertension prediction using a cross-sectional study data in subjects aged over 18 years. They compared three machine learning models of decision tree, random forest, and logistics regression with five-fold cross-validation. Accuracy, positive predictive value (PPV), sensitivity, F-measure, and area, under the receiver operating characteristic curve (AUC), were evaluated for the performance of three machine learning models. In this study, three machine learning models showed similar performances. Similar to our study, AlKaabi et al. found important hypertension determinants including age, gender, education level, employment, tobacco use, physical activity, adequate intakes of fruits and vegetables, abdominal obesity, history of diabetes, history of high cholesterol, and mother’s history high blood pressure [40].

Consistent with our study findings, Iida et al. 2019 [54] also showed the positive association between salt intake and SBP levels in the Japanese elderly subjects aged over 75 years. Body weight was positively associated with higher DBP and higher SBP [54].

In a recent cross-sectional study, overweight/obese subjects showed a higher sodium consumption, compared with non-obese subjects with or without hypertension or overweight/obese subjects without hypertension [55]. This finding indicated the association between higher overall caloric consumption and higher sodium consumption [55]. Moreover, several human studies have shown the association between decreased sodium intake and decreased BP [16,56,57,58]. A low sodium-DASH diet significantly lowered SBP compared with a high sodium-control diet in a randomized clinical trial (RCT) [57]. A random effect meta-analysis found a dose-response association between sodium reduction and a lower risk of hypertension in older subjects, non-white populations, and subjects with a higher BP baseline [56]. 

Our present study observed vitamin C as an important determinant influencing BP. Chen et al. 2002 [59] investigated the effects of serum levels of antioxidant vitamins including vitamin C and α-carotene and β-carotene on BP in 15,317 US adults aged over 20 years involved in in the National Health and Nutrition Examination Survey III (NHANES III). They found the inverse association between serum levels of vitamin C, α-carotene, and β-carotene and BP. Serum levels of vitamin A was positively associated with BP [59]. 

In a 6.1-year follow-up prospective study of 12,245 Chinese adults from the China Health and Nutrition Survey, greater than 227.3 ugRE/day of vitamin A intake was inversely associated with new-onset hypertension compared with less than 227.3 ugRE/day of vitamin A intake. This association indicated that adequately higher vitamin A intake can prevent the risk of hypertension [60]. A nested case-control study from the China Stroke Primary Prevention Trial (CSPPT; 620 cases of first stroke and 620 controls) in adults with hypertension showed that plasma retinol (per 10-μg/dL increment) was associated with a decreased risk of first ischemic stroke by 8% (OR, 0.92; 95% CI 0.86 to 0.98; *p* = 0.012) during the 4.5-year treatment period [61]. Moreover, CSPPT (617 cases of all-cause mortality and 617 controls) found that a 10-μg/dL increase in plasma retinol was associated with lower risk of all-cause mortality in Chinese adults with less than 58.3 μg/dL of plasma retinol (OR, 0.73; 95% CI 0.61 to 0.87), whereas a 10-μg/dL increase in plasma retinol was associated with elevated risk of all-cause mortality in Chinese adults with greater than 58.3 μg/dL of plasma retinol (OR, 1.08; 95% CI 1.01 to 1.16) [62].

In line with the review paper [63], we found calcium as one of the BP determinants. Calcium exerts a role in vasodilation and vasoconstriction of blood vessels. Calcium was associated with lower BP, particularly in adults with hypertension [63]. Calcium can be consumed in dairy products, fishes, and dark leafy green vegetables [63]. 

The strength of this study is the novelty of a deep learning algorithm development in classifying and predicting hypertension based on a large nation-wide sample size. We determined four DNN models with or without energy intake and nutrient intake before or after energy adjustment. Accuracy differences of these four models were explored to identify the influence of nutrient intake on BP prediction. We tried to apply as many relevant BP variables as possible to the deep learning algorithm. The findings of this study provide the key contributors to BP. The deep learning algorithm we developed here enables us to expand further classification and prediction performance of hypertension when data of new persons were entered. 

There are several limitations to take into account when the results are interpreted. We could not find westernized dietary patterns or/and DASH dietary pattern based on the factor analysis. It could be attributable to age, gender, and socio economic and cultural influence. The FFQ for nutrient intake could reflect a self-recall bias. In future studies, a deep learning algorithm should be developed by adding much more data-sized KoGES-Ansan and Ansung follow-up study.

## 6. Conclusions

The deep learning algorithm showed a higher performance compared to the decision tree. Given the best results of dataset I (with energy intake and nutrient intake before energy in-take adjustment) in the DNN, energy intake adjustment is not essential in the DNN with nutrient variables for hypertension classification and prediction. Accurate classification and prediction of the DNN developed in this study might play a critical role in preventing the risk of high BP. Further investigations are required in this important field.

## Figures and Tables

**Table 1:** Food items from food frequency questionnaire.

Food Category (n)	Food Items (n = 103)
**Cereal-rice (4)**	steamed rice, cooked rice with barely, cooked rice with other cereals, mixed multi-grain powder (misugaru/mixed grain powder).
**Cereal-oriental (8)**	ramen, noodle, black bean sauce noodle(chajangmyun), Korean cold noodle/buckwheat noodle, dumpling, white rice cake/rice-cake soup(tteoggug), sticky white rice cake(injeolmi).
**Cereal-western (6)**	bread, bread with red bean/steam bun, other breads (cream bread, castella etc.), pizza/hamburger, cornflake, butter/margarine.
**Vegetables (17)**	green pepper, red pepper leaves, spinach, lettuce, perilla leaf, leek/water dropwort, other vegetables (shepherd’s purse, crown daisy, curled mallow, radish leave etc.), radish (radish soup, boiled radish)/pickled radish, balloon flower/lance asiabell (deodeok), onion, cabbage/cabbage soup, cucumber, bean sprouts, carrot/carrot juice, pumpkin (pumpkin gruel)/pumpkin juice, green pumpkin, vegetable juice/green vegetable juice, bracken/sweet potato stalk, Korean style pickles.
**Fruits (14)**	persimmon/dried persimmon, tangerine, oriental melon/muskmelon, banana, pear, apple/apple juice, orange/orange juice, watermelon, peach/plum, strawberry, grape/grape juice, tomato/tomato juice.
**Potatoes (3)**	potato, sweet potato, starch vermicelli (japchae).
**Legumes (3)**	beans/cooked beans in soy sauce, tofu, stew with soybean paste.
**Kimchi (4)**	kimchi, small radish kimchi(kkakduki), kimchi with liquid, other kimchi (green onion kimchi, leaf mustard kimchi, korean lettuce kimchi etc.)
**Mushrooms (2)**	oyster mushroom, other mushrooms (button mushroom, enokitake, frarant mushroom).
**Seaweeds (2)**	laver, dried kelp/sea mustard.
**Seeds and nuts (2)**	starch jelly, peanut/almond/pine nut.
**Meats (8)**	dog meat, chicken/chicken leg/chicken wing, roast pork (spare rib, pork sirloin etc.), pork belly, steamed pork (soy sauce braised pork, pig trotter, Korean sausage), ham/sausage, roast beef, beef soup/Korean-style braised short rips organ meats.
**Fishes and seafoods (15)**	sashimi (halibut, flat fish, tuna, rockfish etc.), hairtail(cutlassfish), eel, yellow cracker, alaska pollack/frozen pollack/dried pollack, external blue colored fish (mackerel, pacific saury, Spanish mackerel), dried anchovy/stir-fried anchovies, cuttlefish//octopus, tuna/canned tuna, fish paste/crab meat, crab, clam (cockle/short neck clam/clam meat), oyster, shrimp, salt-fermented fish.
**Milk and ** **dairy products (4)**	milk, yogurt, ice cream, cheese.
**Eggs (2)**	egg/quail egg.
**Snack (3)**	marshmallow choco pie(chocopie)/cakes, snacks, candy/chocolate.
**Beverages (7)**	soybean milk, carbonated drinks (cola, sprite), coffee, coffee sugar, coffee cream, green tea, other drinks (sweet rice drink, Korean citron tea[yujacha]).

The value contained in (n) indicate the number of food items.

**Table 2:** Dietary patterns based on factor loading values by factor analysis of 17 food groups rotated component matrix.

Food Groups	Dietary Patterns Factors				
	Dietary Pattern 1	Dietary Pattern 2	Dietary Pattern 3	Dietary Pattern 4	Dietary Pattern 5
Cereal-oriental	0.714				
potatoes	0.621				
Cereal-western	0.578		0.383		
Seeds and nuts	0.563				
Meats		0.807			
Fishes and seafoods		0.671			
Fruits		0.533			
Milk and dairy products			0.612		
Snacks			0.573	−0.306	
Eggs			0.429		
Beverages			0.424		
Seaweeds			0.377		
Mushrooms				0.758	
Vegetables				0.623	
Cereal-rice					0.737
Kimchi					0.611
Legumes					0.409

**Table 3:** Deriving the class of each sample, whether hypertension or not, from three dependent variables.

Variable Name	Variable Explanation	Variable Description	Training Dataset	Testing Dataset
AS1_DrugHtCu	Blood pressure medicine use	1 = No	5802	1466
		2 = Yes	717	164
AS1_BPLIE2S_A	Mean value of 2–time systolic blood pressure measurements (lying position)	mmHg	117.45 (69–211)	117.18 (73–204)
AS1_BPLIE2D_A	Mean value of 2–time diastolic blood pressure measurements (lying position)	mmHg	75.18 (40–125)	75.04 (40–132)
Hypertension	Hypertension	0 = No 1 = Yes	5008 1511	1258 372

**Table 4:** Four datasets according to the energy intake adjustment method.

Dataset	Energy Intake Adjustment Method
Dataset I	with energy intake, and nutrient intake before energy intake adjustment
Dataset II	without energy intake, and with nutrient intake before energy intake adjustment
Dataset III	with energy intake, and with nutrient intake after energy intake adjustment
Dataset IV	without energy intake, and with nutrient intake after energy intake adjustment

**Table 5:** Baseline characteristics of independent variables.

Independent Variables						
Variable Name		Hypertension (n = 1883) (%)	Non-hypertension (n = 6266) (%)	*p*-Value	Training Dataset	Testing Dataset
AS1_Sex				0.096		
	Male	858 (45.6)	2992 (47.7)		3075	775
	Female	1025 (54.4)	3274 (52.3)		3444	855
AS1_Age				<0.001		
	40~49 years	481 (25.5)	3435 (54.8)		3145	771
	50~59 years	579 (30.8)	1533 (24.5)		1684	428
	60~69 years	823 (43.7)	1298 (20.7)		1690	431
AS1_BMI ^†^	BMI(Kg/m^2^)	25.1, 23.6	24.6, 23.1	<0.001	24.67 (14.85–42.00)	24.50 (15.13–36.40)
AS1_Weight ^†^	Weight (Kg)	62.86, 57.40	62.0, 58.0	0.088	63.30 (34–103)	62.84 (36–105)
AS1_WAIST3A ^†^	Waist circumference average value of 3 measurement	86.0, 80.3	81.0, 75.0	<0.001	82.46 (55.66–122.66)	82.21 (57–110)
AS1_B01 ^†^	Energy (Kcal)	1771.0, 1464.0	1849.0, 1545.0	<0.001	1937.29 (127–9985)	1937.00 (230–7034)
AS1_B02 ^†^	Protein (g)	59.0, 45.0	62.0, 48.0	<0.001	66.08 (7–558)	66.30 (7–333)
AS1_B03 ^†^	Fat (g)	25.0, 16.0	29.0, 20.0	<0.001	32.19 (2–357)	32.81 (1–199)
AS1_B04 ^†^	Sugar (carbohydrate, g)	316.0, 272.0	323.0, 279.0	0.014	340.87 (18–1615)	339.35 (35–1184)
AS1_B05 ^†^	Ca (calcium, mg)	397.0, 267.0	433.0, 300.0	<0.001	473.34 (18–2694)	489.25 (51–3226)
AS1_B06 ^†^	P (phosphorus, mg)	930.0, 719.0	971.0, 762.0	<0.001	1019.59 (99–6526)	1028.03 (120–4238)
AS1_B07 ^†^	Fe (iron; mg)	10.0, 7.0	10.0, 8.0	<0.001	10.86 (1–71)	10.90 (1–78)
AS1_B08 ^†^	K (potassium, mg)	2270.0, 1678.0	2351.0, 1770.8	<0.001	2515.75 (193–15,818)	2545.48 (357–13,269)
AS1_B09 ^†^	Vitamin A (retinoids, R.E)	408.0, 263.0	441.0, 293.0	<0.001	530.23 (0–5948)	545.46 (12–6392)
AS1_B10 ^†^	Na (sodium, mg)	2884.0, 2000.0	2917.0, 2092.0	0.142	3163.06 (158–16,760)	3195.08 (160–16,623)
AS1_B11 ^†^	Vitamin B1 (thiamine, mg)	1.0, 1.0	1.0, 1.0	0.016	1.25 (0–10)	1.26 (0–6)
AS1_B12 ^†^	Vitamin B2 (riboflavin, mg)	1.0, 1.0	1.0, 1.0	<0.001	1.03 (0–8	1.05 (0–6)
AS1_B13 ^†^	Vitamin B3 (niacin, nicotinic acid, mg)	14.0, 10.0	15.0, 11.0	<0.001	15.57 (2–170)	15.55 (3–79)
AS1_B14 ^†^	Vitamin C (ascorbic acid, mg)	99.0, 67.0	102.0, 68.0	0.318	125.83 (1–1378)	126.82 (11–987)
AS1_B15 ^†^	Zinc (mg)	8.0, 6.0	8.0, 6.0	<0.001	8.74 (1–112)	8.79 (1–58)
AS1_B16 ^†^	Vitamin B6 (pyridoaxamin, µg)	2.0, 1.0	2.0, 1.0	0.001	1.78 (0–12)	1.78 (0–10)
AS1_B17 ^†^	Folate (µg)	216.0, 156.0	221.0, 165.0	0.010	244.74 (18–1455)	246.50 (23–1835)
AS1_B18 ^†^	Retinol (µg)	44.0, 19.0	58.0, 29.0	<0.001	67.94 (0–742)	70.10 (0–695)
AS1_B19 ^†^	Carotene (µg)	1991.0, 1299.0	2116.0, 1383.0	0.005	2712.82 (3–39,944)	2795.23 (49–40,069)
AS1_B20 ^†^	Ash content (mg)	17.0, 12.0	17.0, 12.0	0.353	21.37 (2–132)	21.71 (3–122)
AS1_B21 ^†^	Fiber (g)	7.0, 5.0	6.0, 5.0	0.203	6.97 (1–38)	7.04 (1–46)
AS1_B23 ^†^	Vitamin E (tocotrienol, µg)	8.0, 5.0	8.0, 6.0	<0.001	9.30 (1–96)	9.38 (1–71)
AS1_B24 ^†^	Cholesterol (mg)	124.0, 64.0	150.0, 87.0	<0.001	175.71 (0–1857)	174.15 (0–1328)
DP1 ^†^	Dietary pattern 1 (cereal-oriental, cereal-western, potatoes, seeds and nuts)	21.0, 8.0	25.0, 10.0	<0.001	45.87 (0–3845)	45.31 (0–1160)
DP2 ^†^	Dietary pattern 2 (fruits, meats, fishes and seafoods)	37.0, 16.0	35.0, 16.0	0.395	136.91 (0–5460)	132.69 (0–2620)
DP3 ^†^	Dietary pattern 3 (snacks, eggs, seaweeds, milk and dairy products, beverages)	74.0, 13.0	114.0, 20.0	<0.001	160.26 (0–1887)	169.05 (0–1971)
DP4 ^†^	Dietary pattern 4 (vegetables, mushrooms)	17.0, 1.0	20.0, 2.0	0.107	44.28 (0–1725)	48.15 (0–1815)
DP5 ^†^	Dietary pattern 5 (cereal-rice, legumes, kimchi)	819.0, 735.0	815.0, 725.8	0.002	846.71 (0–3750)	840.24 (0–2344)

Non-normally distributed values are presented as medians and interquartile ranges. ^†^ Non-parametric values were analyzed by Mann–Whitney U test. AS1_age, age; AS1_BMI, BMI; AS1_B01, energy; AS1_B02, protein; AS1_B03, fat; AS1_B04, sugar (carbohydrate); AS1_B05, Ca (calcium); AS1_B06, P (phosphorus); AS1_B07, Fe (iron); AS1_B08, K (potassium); AS1_B09, vitamin A (retinoids); AS1_B10, Na (sodium); AS1_B11, vitamin B1 (thiamine); AS1_B12, vitamin B2 (riboflavin); AS1_B13, vitamin B3 (niacin, nicotinic acid); AS1_B14, vitamin C (ascorbic acid); AS1_B15, zinc; AS1_B16, vitamin B6 (pyridoaxamine); AS1_B17, folate; AS1_B18, retinol; AS1_B19, carotene; AS1_B20, ash content; AS1_B21, fiber; AS1_B23, vitamin E (tocotrienol); AS1_B24, cholesterol; AS1_Sex, sex; AS1_WAIST3A, waist circumference average value of 3 measurement; AS1_Weight, body weight; DP1, dietary pattern 1 (cereal-oriental, cereal-western, potatoes, seeds and nuts); DP2, dietary pattern 2 (fruits, meats, fishes and seafoods); DP3, dietary pattern 3 (snacks, eggs, seaweeds, milk and dairy products, beverages); DP4, dietary pattern 4 (vegetables, mushrooms); DP5, dietary pattern 5 (cereal-rice, legumes, kimchi).

**Table 6:** Top 10 variable importance in decision tree.

Decision Tree for Variable Importance			
Dataset I	Dataset II	Dataset III	Dataset IV
waist circumference	waist circumference	waist circumference	waist circumference
body mass index	DP3	niacin, nicotinic acid	zinc
age	body mass index	age	body mass index
DP3	age	DP1	niacin, nicotinic acid
retinol	sodium	body mass index	tocotrienol
DP5	sugar (carbohydrate)	zinc	pyridoaxamine
DP1	retinol	retinol	calcium
sodium	DP2	pyridoaxamine	fat
ascorbic acid	DP1	DP3	ash content
DP2	calcium	DP5	carotene

DP1, dietary pattern 1 (cereal-oriental, cereal-western, potatoes, seeds and nuts); DP2, dietary pattern 2 (fruits, meats, fishes and seafoods); DP3, dietary pattern 3 (snacks, eggs, seaweeds, milk and dairy products, beverages); DP4, dietary pattern 4 (vegetables, mushrooms); DP5, dietary pattern 5 (cereal-rice, legumes, kimchi); Dataset I, dataset with energy intake, and nutrient intake before energy intake adjustment; Dataset II, dataset without energy intake, and with nutrient intake before energy intake adjustment; Dataset III, dataset with energy intake, and with nutrient intake after energy intake adjustment; Dataset IV, dataset without energy intake, and with nutrient intake after energy intake adjustment.

**Table 7:** Accuracy of the DNN by varying the number of hidden layers and the number of nodes in each hidden layer.

Accuracy		Hidden Layers			
		2	3	4	5
Nodes	8	0.732	0.735	0.735	0.732
	16	0.718	0.731	0.735	0.729
	24	0.750	0.737	0.734	0.740
	32	0.740	0.728	0.735	0.734
	40	0.726	0.732	0.732	0.732
	48	0.740	0.731	0.731	0.732
	56	0.741	0.737	0.732	0.730
	64	0.740	0.742	0.734	0.733

Accuracy, correctly predicted rate; Hidden layers, layers between the input layer and the output layer; Nodes, size of each hidden layer.

**Table 8:** Performance comparison of DNN and decision tree according to the dataset.

		Dataset I	Dataset II	Dataset III	Dataset IV
DNN	Training error	0.249	0.260	0.256	0.256
	TP	189	166	172	1031
	TN	1034	1040	1040	181
	FP	224	218	218	227
	FN	183	206	200	191
	Sensitivity	0.508	0.446	0.462	0.487
	Specificity	0.822	0.827	0.827	0.820
	F1-score	0.482	0.439	0.451	0.464
	Accuracy	0.750	0.739	0.743	0.743
Decision tree	Training error	0.323	0.315	0.309	0.319
	TP	139	148	168	146
	TN	963	967	957	963
	FP	295	291	301	295
	FN	233	224	204	226
	Sensitivity	0.374	0.398	0.452	0.392
	Specificity	0.766	0.769	0.761	0.766
	F1-score	0.345	0.365	0.400	0.359
	Accuracy	0.676	0.684	0.690	0.680

Accuracy, correctly predicted rate; FN, false negative; FP, false positive; F1-score, combination average of precision and recall; Dataset I, dataset with energy intake, and nutrient intake before energy intake adjustment; Dataset II, dataset without energy intake, and with nutrient intake before energy intake adjustment; Dataset III, dataset with energy intake, and with nutrient intake after energy intake adjustment; Dataset IV, dataset without energy intake, and with nutrient intake after energy intake adjustment; Sensitivity, true positive rate; Specificity, true negative rate; TN, true negative; TP, true positive; Training error, incorrectly predicted rate.

## Data Availability

The authors have no authority over the data, and the data are provided upon request to the Ministry of Health and Welfare.

## Supplementary Materials

The following supporting information can be downloaded at: https://www.mdpi.com/article/10.3390/ijerph192215301/s1, Table S1. Baseline characteristics of independent variables.
