# Supplementary material for: Classification and Prediction on Hypertension with Blood Pressure Determinants in a Deep Learning Algorithm
Source: Int J Environ Res Public Health. 2022 Nov 19;19(22):15301. doi: 10.3390/ijerph192215301 (PMC9690260; doi:10.3390/ijerph192215301)
Supplement: Supplementary file 1 [file ijerph-19-15301-s001.zip › ijerph-1963223-supplementary.pdf]

International Journal of
Environmental Research and Public Health

MDPI

Table S1. Baseline characteristics of independent variables.

Independent Variables						
**Variable Name**		**Hypertension (n = 1,883) (%)**	**Non-hypertension (n = 6,266) (%)**	***p*-Value**	**Training Dataset**	**Testing Dataset**
AS1_Sex				0.096		
	Male	858 (45.6)	2,992 (47.7)		3,075	775
	Female	1,025 (54.4)	3,274 (52.3)		3,444	855
AS1_Age				< 0.001		
	40 ~ 49 years	481 (25.5)	3,435 (54.8)		3,145	771
	50 ~ 59 years	579 (30.8)	1,533 (24.5)		1,684	428
	60 ~ 69 years	823 (43.7)	1,298 (20.7)		1,690	431
AS1_BMI†	BMI(Kg/m²)	25.1, 23.6	24.6, 23.1	< 0.001	24.67 (14.85 – 42.00)	24.50 (15.13 – 36.40)
AS1_Weight†	Weight (Kg)	62.86, 57.40	62.0, 58.0	0.088	63.30 (34 – 103)	62.84 (36 – 105)
AS1_WAIST3A†	Waist circumference average value of 3 measurement	86.0, 80.3	81.0, 75.0	< 0.001	82.46 (55.66 – 122.66)	82.21 (57 – 110)
AS1_EduA	Education			< 0.001		
	Elementary school	875 (46.5)	1,760 (28.1)		2,117	518
	Middle school	397 (21.1)	1,451 (23.2)		1,478	370
	High school	408 (21.7)	2,137 (34.1)		2027	518
	Associate degree	41 (2.2)	254 (4.1)		240	55
	University	144 (7.6)	565 (9.0)		568	141
	Graduate school or higher	18 (1.0)	99 (1.6)		89	28
AS1_Income				< 0.001		
	≥ 500,000won	527 (28.0)	968 (15.4)		1,177	318
	≥ 1,000,000won ~ › 500,000won,	357 (19.0)	902 (14.4)		1,032	227
	≥ 1,500,000won ~ › 1,000,000won,	293 (15.6)	958 (15.3)		1,000	251
	≥ 2,000,000won ~ › 1,500,000won	228 (12.1)	934 (14.9)		931	231
	≥ 3,000,000won ~ › 2,000,000won	239 (12.7)	1,270 (20.3)		1203	306
	≥ 4,000,000won ~ › 3,000,000won	122 (6.5)	707 (11.3)		663	166

	› 6,000,000won› ~ › 4,000,000won	85 (4.5)	390 (6.2)		367	107
	6,000,000won ≥	32 (1.7)	137 (2.2)		145	24
AS1_Drink				0.037		
	Yes	922 (49.0)	2,887 (46.1)		3,043	766
	No (drinking in the past),	125 (6.6)	386 (6.2)		403	108
	No (currently drinking)	836 (44.4)	2,993 (47.8)		3,073	756
AS1_DrDuA				< 0.001		
	≥ 5years	1,011 (53.7)	3,252 (51.9)		3,398	865
	≥ 10years ~ 6years,	63 (3.3)	340 (5.4)		339	64
	≥ 15years ~ 11years	48 (2.5)	234 (3.7)		231	51
	≥ 20years ~ 16years	96 (5.1)	386 (6.2)		391	91
	21years ≥	665 (35.3)	2,054 (32.8)		2,160	559
AS1_TotAlc†	g/day	0.00, 0.00	0.00, 0.00	0.997	0.02 (0 – 22)	0.01 (0 – 22)
AS1_SmokeA				< 0.001		
	Never smoking	1,171 (62.2)	3,689 (58.9)		3,863	967
	Former smoking	338 (18.0)	961 (15.3)		1,062	237
	Sometimes smoking	30 (1.6)	145 (2.3)		126	49
	Smoking habitually	344 (18.3)	1,471 (23.5)		1,438	377
AS1_HvSmAm†	Piece/day	0.00, 0.00	0.00, 0.00	< 0.001	4.28 (0 – 70)	4.51 (0 – 60)
AS1_HvSmDu†	Years	0.00, 0.00	0.00, 0.00	< 0.001	6.50 (0 – 61)	7.16 (0 – 54)
AS1_PhyStb				0.097		
	Not	585 (31.1)	1,916 (30.6)		1,980	521
	› 30minute,	308 (16.4)	922 (14.7)		996	234
	› 60minute ~ ≥ 30 minute	319 (16.9)	1,115 (17.8)		1,154	280
	› 90minute ~ ≥ 60 minute	176 (9.3)	662 (10.6)		662	176
	› 2hours ~ ≥ 90 minute	143 (7.6)	536 (8.6)		545	134
	› 3hours ~ ≥ 2hours	146 (7.8)	528 (8.4)		549	125
	› 4hours ~ ≥ 3hours,	95 (5.0)	296 (4.7)		313	78
	› 5hours ~ ≥ 4hours	34 (1.8)	102 (1.6)		110	26
	≥ 5hours	77 (4.1)	189 (3.0)		210	56
AS1_PhySit				0.464		
	Not	103 (5.5)	313 (5.0)		338	78
	› 30minute,	114 (6.1)	340 (5.4)		364	90
	› 60minute ~ ≥ 30 minute	203 (10.8)	622 (9.9)		667	158
	› 90minute ~ ≥ 60 minute	185 (9.8)	590 (9.4)		605	170

	› 2hours ~ ≥ 90 minute	172 (9.1)	576 (9.2)		613	135
	› 3hours ~ ≥ 2hours	256 (13.6)	805 (12.8)		855	206
	› 4hours ~ ≥ 3hours,	249 (13.2)	840 (13.4)		882	207
	› 5hours ~ ≥ 4hours	131 (7.0)	444 (7.1)		448	127
	≥ 5hours	470 (25.0)	1,736 (27.7)		1,747	459
AS1_PhyActL				0.001		
	Not	94 (5.0)	282 (4.5)		306	70
	› 30minute,	198 (10.5)	524 (8.4)		562	160
	› 60minute ~ ≥ 30minute	293 (15.6)	976 (15.6)		1,032	237
	› 90minute ~ ≥ 60minute	303 (16.1)	865 (13.8)		935	233
	› 2hours ~ ≥ 90minute	198 (10.5)	695 (11.1)		729	164
	› 3hours ~ ≥ 2hours	222 (11.8)	775 (12.4)		811	186
	› 4hours ~ ≥ 3hours,	167 (8.9)	531 (8.5)		537	161
	› 5hours ~ ≥ 4hours	94 (5.0)	325 (5.2)		326	93
	≥ 5hours	314 (16.7)	1,293 (20.6)		1,281	326
AS1_PhyActM				0.104		
	Not	972 (51.6)	3,091 (49.3)		3,243	820
	› 30minute,	279 (14.8)	850 (13.6)		920	209
	› 60minute ~ ≥ 30minute	218 (11.6)	805 (12.8)		810	213
	› 90minute ~ ≥ 60minute	144 (7.6)	545 (8.7)		538	151
	› 2hours ~ ≥ 90minute	65 (3.5)	278 (4.4)		287	56
	› 3hours ~ ≥ 2hours	60 (3.2)	248 (4.0)		242	66
	› 4hours ~ ≥ 3hours,	39 (2.1)	128 (2.0)		139	28
	› 5hours ~ ≥ 4hours	27 (1.4)	83 (1.3)		86	24
	≥ 5hours	79 (4.2)	238 (3.8)		254	63
AS1_PhyActH				< 0.001		
	Not	1,068 (56.7)	3,907 (62.4)		3,971	1,004
	› 30minute,	88 (4.7)	328 (5.2)		333	83
	› 60minute ~ ≥ 30minute	70 (3.7)	286 (4.6)		287	69
	› 90minute ~ ≥ 60minute	60 (3.2)	171 (2.7)		184	47
	› 2hours ~ ≥ 90minute	37 (2.0)	127 (2.0)		133	31
	› 3hours ~ ≥ 2hours	57 (3.0)	185 (3.0)		192	50
	› 4hours ~ ≥ 3hours,	50 (2.7)	133 (2.1)		139	44
	› 5hours ~ ≥ 4hours	58 (3.1)	139 (2.2)		162	35
	≥ 5hours	395 (21.0)	990 (15.8)		1,118	267
AS1_Health				< 0.001		
	Not very healthy	126 (6.7)	197 (3.1)		253	70
	Not healthy	697 (37.0)	1,735 (27.7)		1,954	478

	Normal	599 (31.8)	2,287 (36.5)		2,298	588
	Healthy	441 (23.4)	1,942 (31.0)		1,908	475
	Very healthy	20 (1.1)	105 (1.7)		106	19
AS1_Tied				0.021		
	No	816 (43.3)	2,529 (40.4)		2,674	671
	Yes	1,067 (56.7)	3,737 (59.6)		3,845	959
AS1_SlpAmTm†		7.00, 6.00	7.00, 6.00	< 0.001	6.74 (1-12)	6.70 (1-15)
AS1_SlpAmSf				< 0.001		
	No	588 (31.2)	2,297 (36.7)		2,300	585
	Yes	1,295 (68.8)	3,969 (63.3)		4,219	1,045
AS1_StrPhysJ				< 0.001		
	No	994 (52.8)	2,884 (46.0)		3,098	780
	Yes	889 (47.2)	3,382 (54.0)		3,421	850
AS1_RgMealFqA†		3.00, 3.00	3.00, 3.00	0.001	2.87 (1 – 5)	2.88 (1 – 5)
AS1_FmHtRel1A				< 0.001		
	Father	207 (11.0)	428 (6.8%)		508	127
	Mother	224 (11.9)	510 (8.1%)		595	139
	Brother, Sister	80 (4.2)	105 (1.7%)		138	47
	Other	7 (0.4)	19 (0.3%)		19	7
AS1_FmDmRel1A				0.001		
	Father	45 (2.4)	211 (3.4)		202	54
	Mother	70 (3.7)	363 (5.8)		347	86
	Brother, Sister	69 (3.7)	195 (3.1)		206	58
	Other	10 (0.5)	26 (0.4)		29	7
AS1_FmHeRel1A				0.266		
	Father	28 (1.5)	94 (1.5)		99	23
	Mother	27 (1.4)	139 (2.2)		133	33
	Brother, Sister	18 (1.0)	60 (1.0)		65	13
	Other	6 (0.3)	13 (0.2)		14	5
AS1_FmCvaRel1A				< 0.001		
	Father	113 (6.0)	283 (4.5)		319	77
	Mother	104 (5.5)	246 (3.9)		289	61
	Brother, Sister	33 (1.8)	60 (1.0)		64	29
	Other	14 (0.7)	37 (0.6)		39	12
AS1_FmCvbRel1A				0.959		
	Father	9 (0.5)	30 (0.5)		27	12

	Mother	9 (0.5)	23 (0.4)		29	3
	Brother, Sister	3 (0.2)	10 (0.2)		10	3
	Other	1 (0.1)	2 (0.0)		2	1
AS1_FmCdRel1A				0.060		
	Father	5 (0.3)	10 (0.2)		13	2
	Mother	3 (0.2)	7 (0.1)		10	0
	Brother, Sister	4 (0.2)	3 (0.0)		7	0
	Other	1 (0.1)	0 (0.0)		0	1
AS1_FmChRel1A				0.092		
	Father	0 (0.0)	1 (0.0)		1	0
	Mother	1 (0.1)	8 (0.1)		7	2
	Brother, Sister	2 (0.1)	0 (0.0)		2	0
	Other	0 (0.0)	1 (0.0)		0	1
AS1_FmPvRel1A				0.740		
	Father	0 (0.0)	1 (0.0)		1	0
	Mother	1 (0.1)	5 (0.1)		5	1
	Brother, Sister	1 (0.1)	1 (0.0)		1	1
	Other	0 (0.0)	0 (0.0)		0	0
AS1_FmLpRel1A				0.620		
	Father	2 (0.1)	3 (0.0)		2	3
	Mother	1 (0.1)	9 (0.1)		7	3
	Brother, Sister	1 (0.1)	3 (0.0)		3	1
	Other	0 (0.0)	0 (0.0)		0	0
AS1_B01†	Energy (Kcal)	1,771.0, 1,464.0	1,849.0, 1,545.0	< 0.001	1,937.29 (127 – 9,985)	1,937.00 (230 – 7,034)
AS1_B02†	Protein (g)	59.0, 45.0	62.0, 48.0	< 0.001	66.08 (7 – 558)	66.30 (7 – 333)
AS1_B03†	Fat (g)	25.0, 16.0	29.0, 20.0	< 0.001	32.19 (2 – 357)	32.81 (1 – 199)
AS1_B04†	Sugar (carbohydrate, g)	316.0, 272.0	323.0, 279.0	0.014	340.87 (18 – 1,615)	339.35 (35 – 1,184)
AS1_B05†	Ca (calcium, mg)	397.0, 267.0	433.0, 300.0	< 0.001	473.34 (18 – 2694)	489.25 (51 – 3226)
AS1_B06†	P (phosphorus, mg)	930.0, 719.0	971.0, 762.0	< 0.001	1,019.59 (99 – 6526)	1,028.03 (120 – 4238)
AS1_B07†	Fe (iron; mg)	10.0, 7.0	10.0, 8.0	< 0.001	10.86 (1 – 71)	10.90 (1 – 78)
AS1_B08†	K (potassium, mg)	2,270.0, 1678.0	2,351.0, 1,770.8	< 0.001	2,515.75	2,545.48

					(193 – 15,818)	(357 – 13,269)
AS1_B09†	Vitamin A (retinoids, R.E)	408.0, 263.0	441.0, 293.0	< 0.001	530.23 (0 – 5,948)	545.46 (12 – 6,392)
AS1_B10†	Na (sodium, mg)	2,884.0, 2,000.0	2,917.0, 2,092.0	0.142	3,163.06 (158 – 16,760)	3,195.08 (160 – 16,623)
AS1_B11†	Vitamin B1 (thiamine, mg)	1.0, 1.0	1.0, 1.0	0.016	1.25 (0 – 10)	1.26 (0 – 6)
AS1_B12†	Vitamin B2 (riboflavin, mg)	1.0, 1.0	1.0, 1.0	< 0.001	1.03 (0 – 8	1.05 (0 – 6)
AS1_B13†	Vitamin B3 (niacin, nicotinic acid, mg)	14.0, 10.0	15.0, 11.0	< 0.001	15.57 (2 – 170)	15.55 (3 – 79)
AS1_B14†	Vitamin C (ascorbic acid, mg)	99.0, 67.0	102.0, 68.0	0.318	125.83 (1 – 1,378)	126.82 (11 – 987)
AS1_B15†	Zinc (mg)	8.0, 6.0	8.0, 6.0	< 0.001	8.74 (1 – 112)	8.79 (1 – 58)
AS1_B16†	Vitamin B6 (pyridoaxamin, µg)	2.0, 1.0	2.0, 1.0	0.001	1.78 (0 – 12)	1.78 (0 – 10)
AS1_B17†	Folate (µg)	216.0, 156.0	221.0, 165.0	0.010	244.74 (18 – 1,455)	246.50 (23 – 1,835)
AS1_B18†	Retinol (µg)	44.0, 19.0	58.0, 29.0	< 0.001	67.94 (0 – 742)	70.10 (0 – 695)
AS1_B19†	Carotene (µg)	1,991.0, 1,299.0	2,116.0, 1,383.0	0.005	2,712.82 (3 – 39,944)	2,795.23 (49 – 40,069)
AS1_B20†	Ash content (mg)	17.0, 12.0	17.0, 12.0	0.353	21.37 (2 – 132)	21.71 (3 – 122)
AS1_B21†	Fiber (g)	7.0, 5.0	6.0, 5.0	0.203	6.97 (1 – 38)	7.04 (1 – 46)
AS1_B23†	Vitamin E (tocotrienol, µg)	8.0, 5.0	8.0, 6.0	< 0.001	9.30 (1 – 96)	9.38 (1 – 71)
AS1_B24†	Cholesterol (mg)	124.0, 64.0	150.0, 87.0	< 0.001	175.71 (0 – 1857)	174.15 (0 – 1,328)
DP1†	Dietary pattern 1 (cereal-oriental, cereal-western, potatoes, seeds and nuts)	21.0, 8.0	25.0, 10.0	< 0.001	45.87 (0 – 3,845)	45.31 (0 – 1,160)
DP2†	Dietary pattern 2 (fruits, meats, fishes and seafoods)	37.0, 16.0	35.0, 16.0	0.395	136.91 (0 – 5,460)	132.69 (0 – 2,620)
DP3†	Dietary pattern 3	74.0, 13.0	114.0, 20.0	< 0.001	160.26 (0 – 1,887)	169.05 (0 – 1,971)

	(snacks, eggs, seaweeds, milk and dairy products, beverages)					
DP4†	Dietary pattern 4 (vegetables, mushrooms)	17.0, 1.0	20.0, 2.0	0.107	44.28 (0 – 1,725)	48.15 (0 – 1,815)
DP5†	Dietary pattern 5 (cereal-rice, legumes, kimchi)	819.0, 735.0	815.0, 725.8	0.002	846.71 (0 – 3,750)	840.24 (0 – 2,344)

Non-normally distributed values are presented as medians and interquartile ranges. † Non-parametric values were analyzed by Mann-Whitney U test. **AS1_age**, age; **AS1_BMI**, BMI; AS1_B01, energy; **AS1_B02**, protein; **AS1_B03**, fat; **AS1_B04**, sugar (carbohydrate); **AS1_B05**, Ca (calcium); **AS1_B06**, P (phosphorus); **AS1_B07**, Fe (iron); **AS1_B08**, K (potassium); **AS1_B09**, vitamin A (retinoids); **AS1_B10**, Na (sodium); **AS1_B11**, vitamin B1 (thiamine); **AS1_B12**, vitamin B2 (riboflavin); **AS1_B13**, vitamin B3 (niacin, nicotinic acid); **AS1_B14**, vitamin C (ascorbic acid); **AS1_B15**, zinc; **AS1_B16**, vitamin B6 (pyridoaxamine); **AS1_B17**, folate; AS1_B18, retinol; **AS1_B19**, carotene; **AS1_B20**, ash content; **AS1_B21**, fiber; **AS1_B23**, vitamin E (tocotrienol); **AS1_B24**, cholesterol; **AS1_DrDuA**, drinking period; **AS1_Drink**, can't drink or don't drink from the beginning (for religious reasons); **AS1_EduA**, education; **AS1_FmCdRel1A**, relationship between family members and subjects diagnosed with coronary artery disease (angina pectoris, arteriosclerosis) in the past; **AS1_FmChRel1A**, relationship between family members and subjects diagnosed with congestive heart failure in the past; **AS1_FmCvaRel1A**, relationship between family members who have been diagnosed with a stroke(paralysis) in the past and the subject; **AS1_FmCvbRel1A**, relationship between family members and subjects diagnosed with cerebrovascular(excluding stroke) disease in the past; **AS1_FmDmRel1A**, relationship between family members who have been diagnosed with diabetes in the past and the subject; **AS1_FmHeRel1A**, relationship between family members who have been diagnosed with heart disease in the past; **AS1_FmHtRel1A**, relationship between family members and subjects diagnosed with hypertension in the past; **AS1_FmLpRel1A**, relationship between family members and subjects diagnosed with hyperlipidemia in the past; **AS1_FmPvRel1A**, relationship between family members and subjects diagnosed with peripheral vascular disease in the past; **AS1_Health**, health status; **AS1_HvSmAm**, the amount of smoking per day; **AS1_HvSmDu**, smoking period; **AS1_Income**, monthly income; **AS1_PhyActH**, physical activity time/day (high activity); **AS1_PhyActL**, physical activity time/day (light activity); **AS1_PhyActM**, physical activity time/day (middle activity); **AS1_PhySit**, physical activity time/day (sedentary lifestyle); **AS1_PhyStb**, physical activity time/day (stable state); **AS1_RgMealFqA**, the number of meals a day; **AS1_Sex**, sex; **AS1_SlpAmSf**, enough time to sleep; **AS1_SlpAmTm**, sleeping time; **AS1_SmokeA**, smoking status; **AS1_StrPhysJ**, physical sign: the body feels drowsy and tires easily; **AS1_Tied**, feel tired these days; AS1_TotAlc, the total amount of alcohol consumption; **AS1_WAIST3A**, waist circumference average value of 3 measurement; **AS1_Weight**, body weight; **DP1**, dietary pattern 1 (cereal-oriental, cereal-western, potatoes, seeds and nuts); **DP2**, dietary pattern 2 (fruits, meats, fishes and seafoods); **DP3**, dietary pattern 3 (snacks, eggs, seaweeds, milk and dairy products, beverages); **DP4**, dietary pattern 4 (vegetables, mushrooms); **DP5**, dietary pattern 5 (cereal-rice, legumes, kimchi).
